# Deep Learning-Enabled Virtual Multiplexed Immunostaining of Label-Free Tissue for Vascular Invasion Assessment

**DOI:** 10.34133/bmef.0226

**Published:** 2026-02-10

**Authors:** Yijie Zhang, Çağatay Işıl, Xilin Yang, Yuzhu Li, Anna Elia, Karine Atlan, William Dean Wallace, Nir Pillar, Aydogan Ozcan

**Affiliations:** ^1^ Electrical and Computer Engineering Department, University of California, Los Angeles, CA 90095, USA.; ^2^ Bioengineering Department, University of California, Los Angeles, CA 90095, USA.; ^3^ California NanoSystems Institute (CNSI), University of California, Los Angeles, CA 90095, USA.; ^4^ Department of Pathology, Hadassah Hebrew University Medical Center, Jerusalem 91120, Israel.; ^5^ Department of Pathology, Keck School of Medicine, University of Southern California, Los Angeles, CA 90033, USA.; ^6^ Department of Surgery, University of California, Los Angeles, CA 90095, USA.

## Abstract

**Objective:** We report the development and validation of a deep learning-based virtual multiplexed immunostaining method for label-free tissue, enabling the simultaneous generation of ERG (ETS-related gene), PanCK (pan-cytokeratin), and hematoxylin and eosin (H&E) images for vascular invasion assessment. **Impact Statement:** This work delivers routine laboratory-compatible virtual multiplexed immunohistochemistry (mIHC) that reproduces ERG, PanCK, and H&E on the same tissue section without chemical staining. It addresses the cost, labor, tissue loss, and section-to-section variability of conventional IHC, as well as the practical unavailability of mIHC in most pathology laboratories, thereby improving accuracy and efficiency in assessing vascular invasion. **Introduction:** Traditional IHC requires one tissue section per stain, exhibits section-to-section variability, and incurs high costs and laborious staining procedures. While mIHC techniques enable simultaneous staining with multiple antibodies on a single slide, they are more tedious to perform and are currently unavailable in routine pathology laboratories. Here, we present a deep learning-based virtual multiplexed immunostaining framework that simultaneously generates ERG and PanCK, in addition to H&E virtual staining, enabling the accurate localization and interpretation of vascular invasion in thyroid cancers. **Methods:** This virtual mIHC technique is based on the autofluorescence microscopy images of label-free tissue sections, and its output images closely match the histochemical staining counterparts (ERG, PanCK, and H&E) of the same tissue sections. **Results:** Blind evaluation by board-certified pathologists demonstrated that virtual mIHC staining achieved high concordance with the histochemical staining results, accurately highlighting epithelial and endothelial cells. Virtual mIHC conducted on the same tissue section also allowed the identification and localization of small vessel invasion. **Conclusion:** This virtual mIHC approach can substantially improve diagnostic accuracy and efficiency in the histopathological evaluation of vascular invasion, potentially eliminating the need for traditional staining protocols and mitigating issues related to tissue loss and heterogeneity.

## Introduction

The introduction of immunohistochemistry (IHC) into routine pathology practice in the early 1990s marked a significant advancement in diagnostic precision. By enabling the visualization of specific proteins within tissue sections, IHC allowed pathologists to move beyond morphological assessment and incorporate molecular information into their diagnoses [1]. In routine clinical pathology, IHC typically requires one slide per antibody stain, as each marker is applied to a separate tissue section. This one-marker-per-slide approach results in increased tissue consumption, especially critical in small biopsies. It also introduces variability due to section-to-section differences and increases the workload and costs associated with slide preparation, staining, and interpretation.

While standard IHC methodology enables labeling of a single marker per tissue section, multiplexed IHC (mIHC) technologies, introduced over the last decade, allow simultaneous staining of a tissue section with multiple antibodies [2]. These multiplexed histochemical staining approaches, however, demand advanced imaging and analysis tools, are expensive, and have a very slow turnaround time. None of these methodologies has received clinical approval as diagnostic assays. To overcome these limitations while maintaining compatibility with existing clinical workflows, pathology laboratories have adopted duplex IHC staining—a method that uses 2 antibodies, each detected by a distinct chromogen through secondary antibodies. This approach enables spatial localization of 2 biomarkers on the same tissue slide using standard IHC protocols routinely employed in clinical practice. One such example is the identification of vascular invasion, defined by the presence of tumor cells within blood vessels or lymphatic vessels. Vascular invasion represents a critical step in the metastatic cascade, facilitating both lymphatic spread and systemic dissemination of cancer cells. Its prognostic significance has been well established across a range of solid tumors, including breast [3], colon [4], bladder [5], lung [6], thyroid carcinoma [7], and melanoma [8]. The identification of vascular invasion may influence clinical decision-making and support the implementation of more aggressive approaches, such as adjuvant chemotherapy or radiation.

**Fig. 1.**
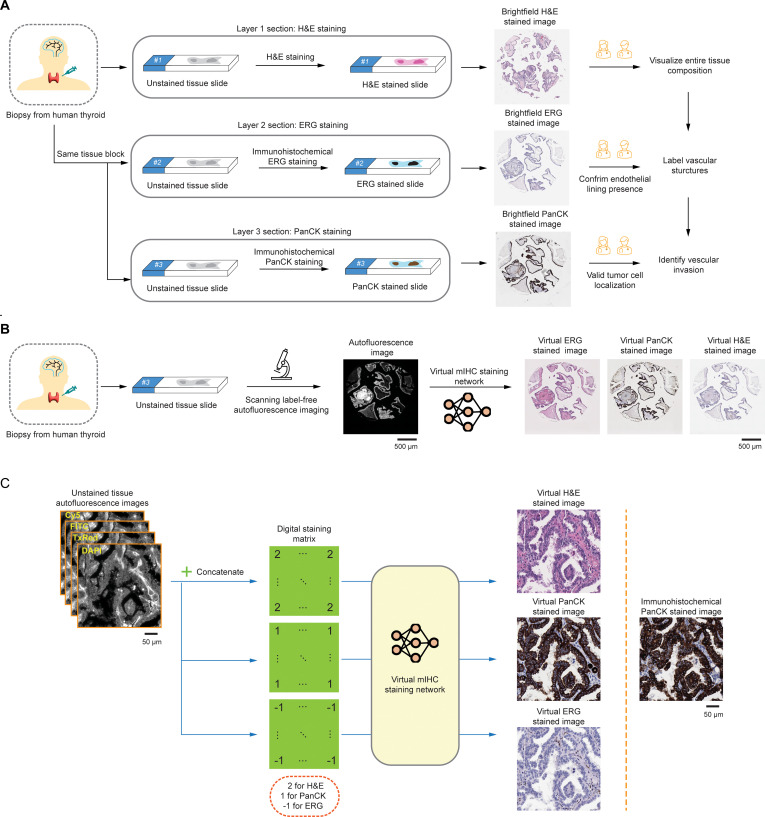
Virtual multiplexed IHC (mIHC) staining of label-free thyroid tissue for vascular invasion assessment. (A) Clinical workflow for assessing vascular invasion in human thyroid tissue. (B) Workflow of virtual mIHC staining using label-free AF imaging of unstained thyroid tissue. (C) Virtual staining network using AF inputs and a DSM to generate BF-equivalent images for H&E, PanCK, and ERG stains.

The identification of vascular invasion on hematoxylin and eosin (H&E)-stained sections is an important component of histopathologic evaluation of tumors, but it can be challenging due to artifacts such as tissue retraction, cracks, or pseudovascular spaces [4] that may mimic true vascular structures. While IHC staining for endothelial markers such as ERG, CD31, or D2-40 can help, they do not provide information about the nature of the intravascular cells. In such cases, uncertainty may arise as to whether the cells within the vessel are malignant or represent benign cells, such as histiocytes. To address this limitation, duplex IHC staining, which combines an endothelial marker (e.g., ERG) with an epithelial marker (e.g., cytokeratin), can allow for the simultaneous visualization of both vascular architecture and tumor cell localization, thereby improving diagnostic accuracy and reducing the risk of misinterpretation [9,10]. However, the duplex IHC technique is available only in large pathology laboratories, whereas most small- to medium-sized laboratories typically perform a single IHC stain per slide, as illustrated in Fig. 1A. In certain cases where vascular invasion is suspected on H&E-stained sections and IHC is ordered to confirm the presence of tumor cells within vascular structures, the area of interest may be lost in deeper tissue levels (particularly when involving small-caliber vessels) due to sectioning variability or tissue dropout. This challenge underscores the importance of performing immunostaining on sections cut at the same tissue level as the original H&E slide to ensure accurate localization and interpretation of the suspicious focus.

Recently, virtual histological staining of label-free tissue sections has been successfully demonstrated for various stain types [11–23]. These techniques computationally transform microscopy images of unstained tissue into digitally stained images that closely match conventional chemically stained images of the same tissue samples. Advances in deep learning methods and powerful computing hardware have significantly accelerated the development of these virtual staining approaches [16,18–21,24–33], effectively applying them to common stains such as H&E [11] and IHC [34]. Recent advancements in multiplexed virtual staining have enabled the visualization of multiple stains (e.g., H&E, Masson’s trichrome, and Jones’ silver) on the same tissue area using a single trained model [35]. This multiplexing approach was further extended to concurrently learn cross-modality image transformations within a single neural network, simultaneously generating virtual birefringence imaging and virtual Congo red staining of amyloid deposits from the same label-free autofluorescence (AF) inputs [36].

Here, we present a deep learning-based virtual multiplexed immunostaining approach designed to simultaneously generate virtual H&E and duplex IHC staining on label-free thyroid tissue sections, enabling accurate localization and interpretation of vascular invasion in thyroid cancers. As illustrated in Fig. 1B, our method utilizes conditional generative adversarial networks (cGANs) to rapidly convert AF microscopy images of unstained tissue slides into virtually stained images (H&E, ERG, and PanCK), matching the corresponding histochemically stained images of the same tissue section. To accomplish multiplexed staining, we integrated a digital staining matrix (DSM) as an additional input channel alongside AF images within the cGAN framework, as depicted in Fig. 1C. To validate this approach, we trained our virtual staining network using a dataset mixed with paired AF images of label-free tissue microarray (TMA) cores and their histochemically stained counterparts (H&E, ERG, and PanCK). After its training, our virtual mIHC staining network successfully generated virtual H&E-, ERG-, and PanCK-stained images from label-free test TMA cores, never used during training. The resulting virtual IHC images (ERG and PanCK) and their corresponding histochemically stained ground truth were randomly shuffled and blindly evaluated by board-certified pathologists. Four pathologists independently affirmed the non-inferior quality of the digitally generated virtual duplex IHC images compared to their histochemical staining counterparts, noting a high concordance with the ground-truth images. Additionally, a pathologist successfully identified and localized vascular invasion using the generated virtual H&E and duplex IHC images. The presented virtual mIHC staining approach effectively eliminates the need for H&E staining followed by dual IHC staining procedures on serial tissue sections, circumventing limitations such as tissue dropout and sectioning variability, and it has the potential to accelerate and enhance the diagnostic accuracy for vascular invasion in routine histopathological evaluation.

## Results

### Virtual mIHC staining of label-free tissue

Initially, we captured label-free AF microscopy images from TMA cores of human thyroid tissue samples. Subsequently, these samples were separately subjected to conventional histopathological staining processes, including H&E and IHC staining with ERG and PanCK antibodies. The resulting stained sections were scanned using brightfield (BF) microscopy to obtain BF images of stained TMA cores, serving as ground-truth references for both the training and testing phases. Each AF image of label-free TMA core was paired and finely registered with its corresponding BF image after each staining process (one staining modality per section), resulting in 3 distinct datasets corresponding to the H&E, ERG, and PanCK stains; see the Methods section for details. Our virtual mIHC staining model leveraged a cGAN, incorporating a DSM concatenated with the input AF channels. This DSM, matching the pixel dimensions of the input images, determined the desired staining output at the pixel level, encoded as “2” for H&E, “1” for PanCK, and “−1” for ERG staining. During the training process, all 3 datasets were mixed to ensure that the network learned accurate transformations from label-free AF images to the respective virtual staining channel. Further details on data acquisition, preprocessing, and network architecture are presented in the Methods section.

**Fig. 2.**
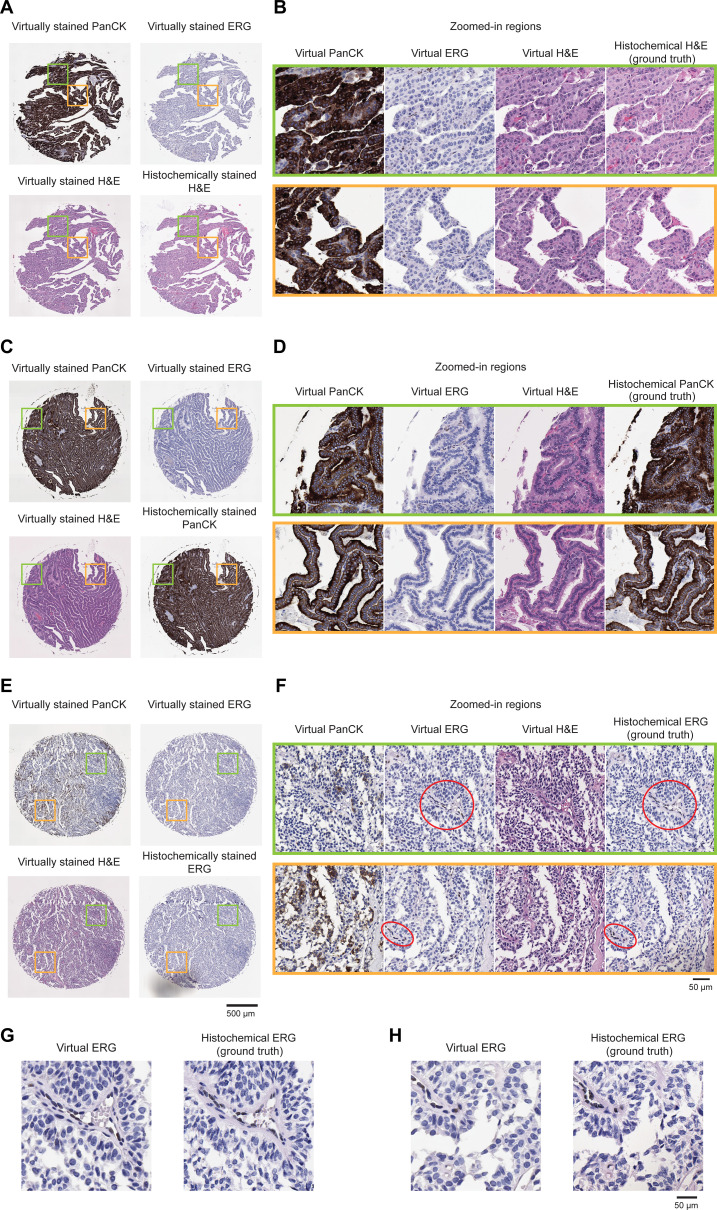
Visual comparison between the virtual multiplexed stains of label-free thyroid sample and their histochemically stained counterparts. (A, C, and E) Virtually stained H&E, PanCK, and ERG images of 3 representative label-free thyroid TMA cores. For each core, the corresponding histochemically or immunohistochemically stained reference image (H&E, PanCK, or ERG) is also shown. (B, D, and F) Zoomed-in views of the selected regions (green and orange boxes) in (A), (C), and (E), highlighting detailed comparisons between virtual and histochemical staining for each stain type. The H&E virtual slides closely reproduce the tissue morphology and color contrast seen in the histochemical reference, enabling the evaluation of cellular size and shape, nuclear chromatin texture, and stromal organization. Virtual IHC staining for PanCK demonstrates cytoplasmic staining of epithelial cells, allowing the evaluation of epithelial distribution and integrity. ERG IHC staining shows a typical nuclear pattern, as highlighted in red circled regions in (F), enabling the assessment of vascular and endothelial structures. (G and H) Zoomed-in region of interest of the virtual ERG and their corresponding histochemical ground truth from (F) present the clear DAB nuclear labeling. The virtually stained sections convincingly highlighted several endothelial cells (flattened cells lining vascular spaces containing scattered red blood cells) with strong nuclear staining, detecting a greater number of positive cells than the corresponding immunohistochemical stains.

Following its training, the virtual mIHC staining model was evaluated in a blinded manner using label-free AF images from 30 previously unseen TMA cores specifically reserved for testing. Among these 30 cores, 12 were subsequently stained with H&E, 12 with PanCK, and 6 with ERG to acquire ground-truth histochemically stained images—only used for comparison purposes. Across all 3 staining modalities, the generated virtual staining images exhibited a good agreement with the corresponding ground-truth images, as illustrated in Fig. 2. This figure provides side-by-side visual comparisons between the virtually stained images produced by our model and their histochemically stained counterparts. Specifically, Fig. 2A, C, and E demonstrates representative examples from 3 distinct patient-based TMA cores (not used for model training), each subjected to H&E, PanCK, or ERG staining after the AF imaging. Detailed comparisons of the zoomed-in regions for each staining modality are presented in Fig. 2B, D, and F, highlighting the quality of the virtual staining results relative to the histochemically stained ground-truth images. The virtual H&E slides effectively replicate the tissue morphology and color contrast observed in the histochemical reference, allowing for the evaluation of cell size and shape, nuclear chromatin texture, and stromal organization. Virtual IHC staining for PanCK shows cytoplasmic labeling of epithelial cells, enabling epithelial distribution and structural integrity. As highlighted by the red circled regions in Fig. 2F and the zoomed-in views in Fig. 2G and H, the virtual ERG IHC staining exhibits nuclear patterns, permitting the evaluation of vascular and endothelial components, demonstrating high concordance with the histochemical ground-truth images.

**Fig. 3.**
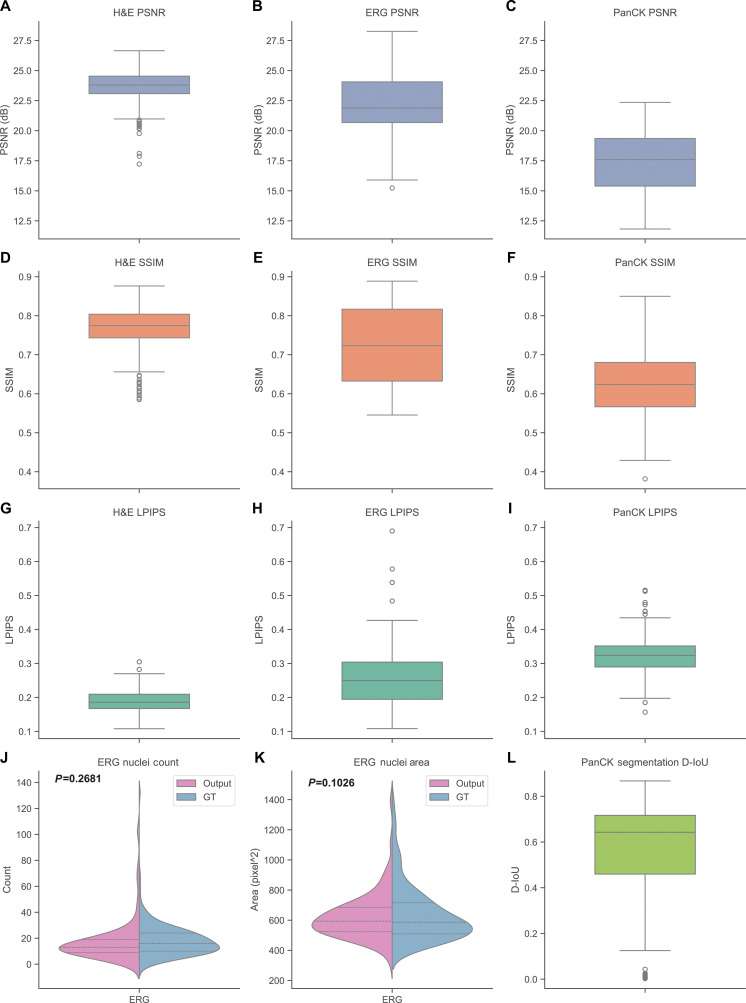
Quantitative evaluation of virtual staining fidelity. (A to I) Image-level fidelity metrics comparing virtual staining results with their corresponding histochemical ground-truth images across H&E, ERG, and PanCK stains. Results are reported using 3 standard metrics—PSNR (A to C), SSIM (D to F), and LPIPS (G to I)—computed on held-out test sets (390 H&E pairs, 197 ERG pairs, and 149 PanCK pairs). Higher PSNR and SSIM values and lower LPIPS values indicate strong structural and perceptual similarity between virtual and histochemical stains. (J and K) ERG nuclear localization analysis. ERG-positive endothelial nuclei were extracted from both virtual and histochemical images using color deconvolution of the DAB channel (see Methods and Fig. S4). Histograms of nuclei count (J) and nuclei area (K) between virtual and ground-truth ERG stains are displayed. (L) PanCK epithelial localization analysis. Epithelial cell masks were extracted from virtual and histochemical PanCK images, and D-IoU was computed between the 2 segmentation masks. High D-IoU values indicate strong concordance of epithelial staining patterns across virtual and histochemical images.

In addition to the qualitative evaluations, we quantitatively assessed the fidelity of the virtually stained H&E, ERG, and PanCK images using 3 standard image-level metrics: peak signal-to-noise ratio (PSNR), structural similarity index (SSIM) [37], and learned perceptual image patch similarity (LPIPS) [38]. These metrics were computed on held-out test sets comprising 390 H&E image pairs from 12 unique TMA cores, 197 ERG pairs from 6 TMA cores, and 149 PanCK pairs from another 6 TMA cores. As shown in Fig. 3A to I, the virtually stained images exhibit high structural similarity and perceptual consistency with their histochemical counterparts across all 3 types of stains.

For the ERG staining, we further evaluated the fidelity of nuclear localization. Using color deconvolution to extract the DAB (3,3′-diaminobenzidine) channel (see Methods and Fig. S4), we identified ERG-positive endothelial nuclei in both the virtually stained and histochemically stained images and compared their distributions. The histograms of nuclei counts and areas shown in Fig. 3J and K demonstrate a strong agreement between the two, indicating that the virtual ERG stain correctly highlights endothelial structures surrounding blood vessels. Similarly, for PanCK, we extracted epithelial cell masks from both the virtual and histochemical images and computed the down-sampled intersection over union (D-IoU) between the 2 segmentation masks (Fig. 3l). The high D-IoU values confirm that the virtual PanCK stain accurately reproduces the epithelial cell distributions observed in the ground-truth images.

Together, these quantitative assessments corroborate the visual comparisons and demonstrate that the virtual staining model achieves high fidelity across H&E, ERG, and PanCK stains.

### Evaluations of virtual mIHC (PanCK and ERG) staining by board-certified pathologists

**Fig. 4.**
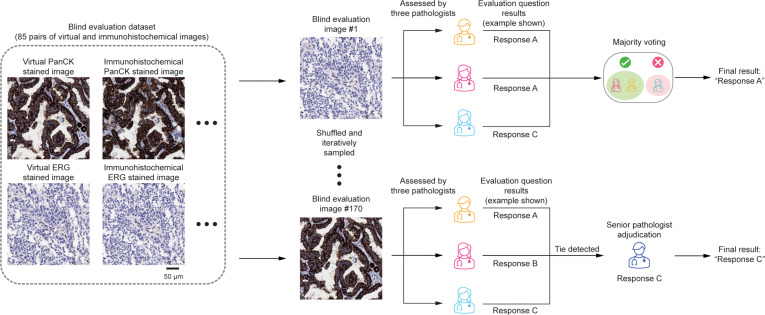
Overview of the study design incorporating board-certified pathologists’ review. A total of 85 pairs of virtual and immunohistochemically stained images (170 images in total) were randomly shuffled and iteratively presented to 3 pathologists in a blinded fashion. Each image was independently assessed, and responses to predefined evaluation questions were recorded. The final evaluation result was determined by majority voting. In cases where a tie occurred, a fourth senior pathologist was consulted to adjudicate and provide the final decision.

To further validate the efficacy of our deep learning-based mIHC virtual staining approach, we conducted a study involving 39 pairs of virtual ERG images with their corresponding histochemically stained ground-truth images, as well as 46 pairs of virtual PanCK images with their corresponding ground-truth images. The image pairs (each image with 2,000 × 2,000 pixels) were sampled from 6 ERG-stained TMA cores and 6 PanCK-stained TMA cores reserved for testing. As illustrated in Fig. 4, these 85 image pairs (totaling 170 images) were randomized and sequentially presented in a blinded manner to 3 board-certified pathologists (A.E., K.A., and N.P.). Each pathologist evaluated the images based on 4 standardized questions:

1. What is the staining pattern? (“n” for nuclear or “c” for cytoplasmic)
2. How would you rate the staining intensity? (Scale: 1 for weak, 2 for moderate, and 3 for strong)
3. Indicate your agreement with the statement:

• For nuclear staining patterns: “The stain highlights blood vessels.”
• For cytoplasmic staining patterns: “The stain highlights epithelial cells.”

4. Indicate your agreement with the statement:

• For nuclear staining patterns: “The stain highlights cells other than endothelial cells.”
• For cytoplasmic staining patterns: “The stain highlights cells other than epithelial cells.”

For questions 3 and 4, responses were recorded on a 3-point scale: 1 for “disagree”, 2 for “equivocal”, and 3 for “agree”. Additionally, if a pathologist was unable to confidently respond to any question, their answer was recorded as “cannot determine”. After obtaining evaluations from the 3 pathologists, we applied a majority voting strategy to determine the final response for each image, as depicted in Fig. 4. If all 3 responses differed (resulting in a tie), an additional evaluation was performed by a senior fourth pathologist (W.D.W.) to provide a final adjudicated response.

**Fig. 5.**
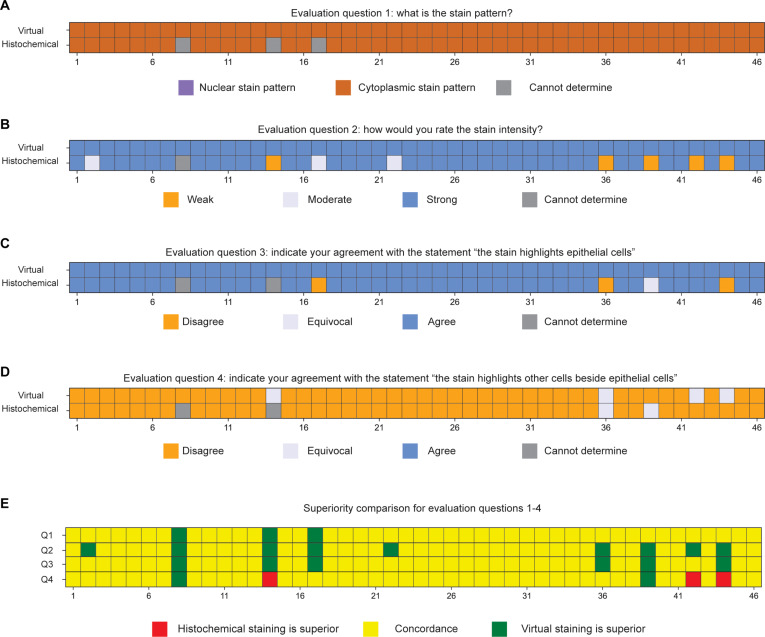
Visualization of pathological evaluation results for PanCK stain. (A to D) Evaluation results for questions 1 to 4 (as defined in Fig. 4) for each virtually and histochemically stained PanCK image pair. The questions assessed (A) stain pattern, (B) stain intensity, (C) agreement on whether the stain highlights epithelial cells, and (D) whether the stain highlights non-epithelial cells. (E) Superiority comparison based on evaluation questions 1 to 4, indicating whether the virtually stained or histochemically stained PanCK image was rated superior, or if the responses were concordant.

The evaluation results for the 46 pairs of virtual and histochemical PanCK-stained images, assessed according to predefined evaluation questions, are summarized in Fig. 5A to D. These results illustrate strong concordance between virtually stained PanCK images and their histochemically stained counterparts across multiple evaluation dimensions. Specifically, the pathologists consistently recognized both virtual and histochemically stained PanCK images as having a robust cytoplasmic staining pattern, effectively highlighting the targeted epithelial cells without significant staining of off-target cells. To better compare the virtual PanCK images with their histochemical counterparts, Fig. 5E presents a statistical superiority analysis based on the pathologists’ responses to the 4 evaluation questions. The virtually stained images consistently matched the histochemical staining results, and notably, in some instances, virtual staining demonstrated superior performance, particularly regarding consistent stain intensity and targeted epithelial cell highlighting.

**Fig. 6.**
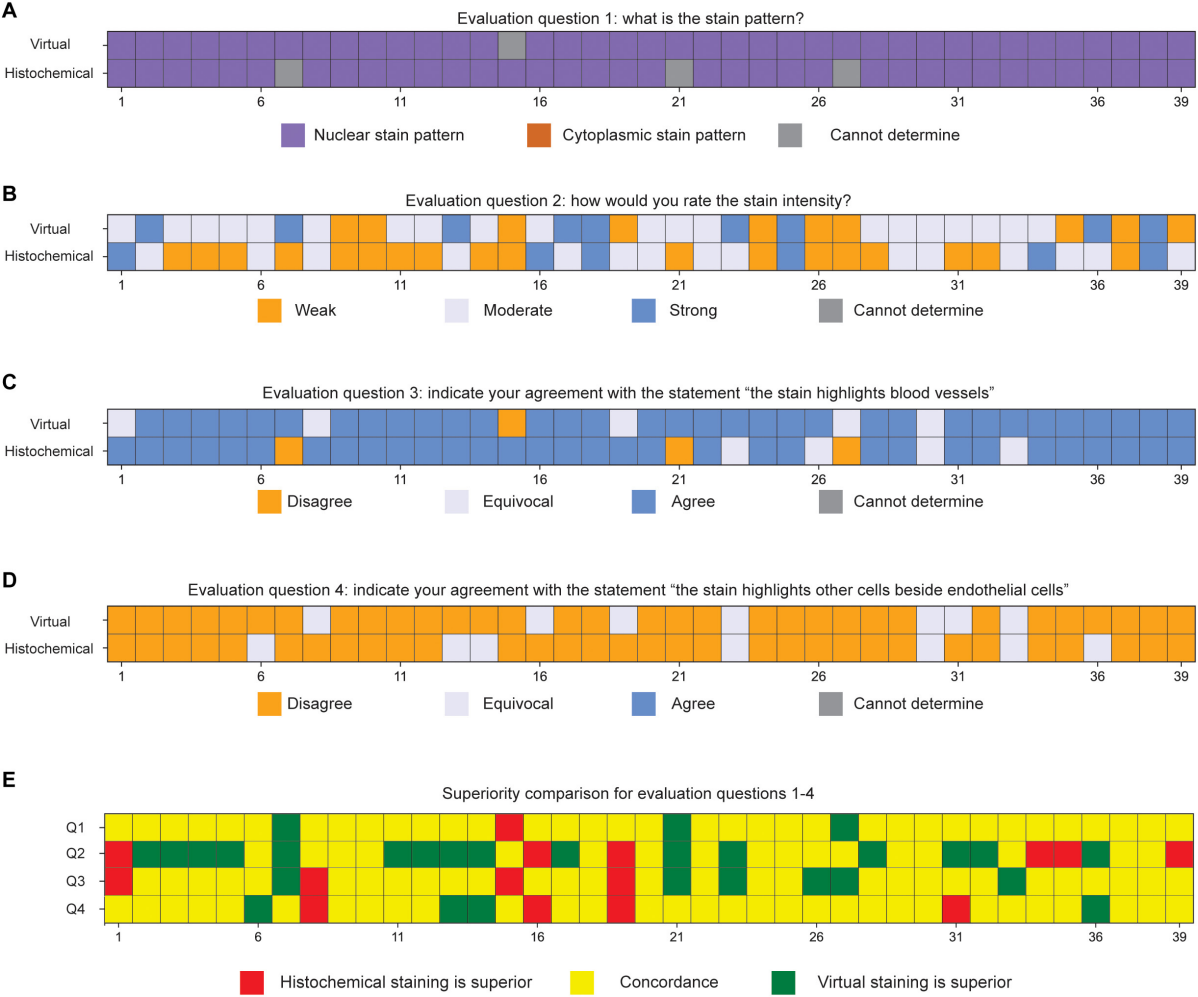
Visualization of pathological evaluation results for ERG stain. (A to D) Evaluation outcomes for questions 1 to 4 (as defined in Fig. 4) for each virtual and histochemically stained ERG image pair, shown per case. Responses include (A) stain pattern, (B) stain intensity, (C) agreement on endothelial cell highlighting, and (D) agreement on staining of non-endothelial cells. (E) Superiority comparison for each evaluation question across all cases, indicating whether the virtually stained or histochemically stained image was rated superior, or if the results were concordant.

Similarly, the evaluation results for the 39 pairs of ERG-stained images are presented in Fig. 6A to D. The majority of pathologists recognized both the virtual and histochemical ERG-stained images as having a nuclear staining pattern, consistently highlighting targeted endothelial cells associated with blood vessels. Figure 6E provides a statistical superiority analysis, again indicating a high level of agreement between the virtual and histochemically stained tissue images across all evaluation aspects. Notably, the virtual ERG staining occasionally exhibited superior performance, particularly regarding staining specificity with minimal background staining. This advantage potentially arises from technical limitations inherent in histochemical ERG staining, such as nonspecific antibody binding and suboptimal antibody quality, which can sometimes result in inadequate visualization of endothelial cells. Thus, the virtual mIHC staining method demonstrates superior reliability in consistently labeling endothelial cells.

To further assess the relevant histopathological features highlighted in this comparison, a board-certified pathologist (N.P.) reviewed virtual and histochemical ERG images for 3 representative cases (#7, #19, and #21), as shown in Fig. S1. In cases #7 and #21, the virtually stained sections convincingly highlighted several endothelial cells (flattened cells lining vascular spaces containing scattered red blood cells) with strong nuclear staining, while the corresponding histochemical stains failed to detect ERG-positive endothelial cells. Conversely, in case #19, multiple endothelial cells displayed positive immunohistochemical ERG staining, whereas the virtual staining highlighted only occasional ERG-positive cells.

**Fig. 7.**
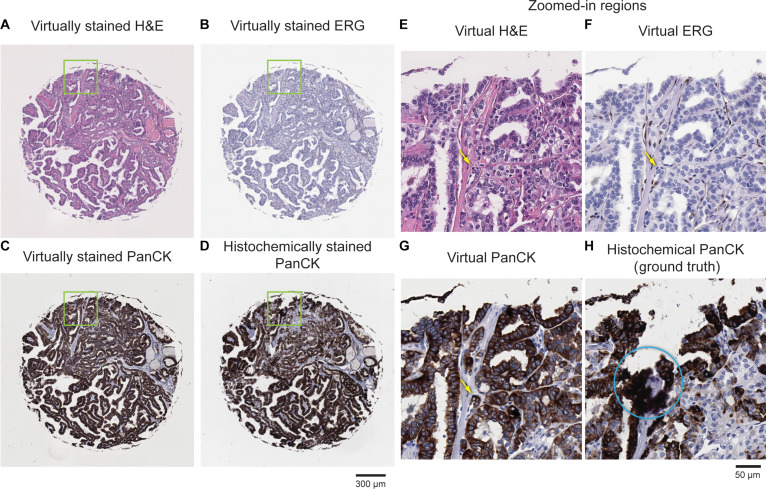
Example of virtual mIHC staining used to identify vascular invasion. (A to C) Virtually stained H&E, ERG, and PanCK images of a label-free thyroid TMA core from a patient diagnosed with metastatic disease. (D) Corresponding histochemically stained PanCK image of the same TMA core. (E to H) Zoomed-in views of the regions outlined in (A) to (D). As identified by a board-certified pathologist (N.P.), the yellow arrows indicate a region of vascular invasion, and the blue circled region indicates an area with nonspecific staining, as part of the histochemical PanCK.

These pathological evaluation results, both quantitatively and qualitatively, demonstrate that the virtually stained PanCK and ERG images effectively highlight target epithelial cells and endothelial cells, respectively. To further illustrate how our virtual mIHC staining assists in identifying vascular invasion, Fig. 7A to C presents virtually stained H&E, PanCK, and ERG images of a representative TMA core from a patient diagnosed with papillary thyroid carcinoma and distant metastasis. The corresponding histochemically stained PanCK ground-truth image is also displayed in Fig. 7D. The yellow arrows in the zoomed-in images highlight endothelial cells (Fig. 7F) and epithelial cells (Fig. 7G) within the vessel lumen, indicative of vascular invasion, as identified by a board-certified pathologist (N.P.). In contrast, the histochemically stained PanCK image exhibited some staining artifacts, as illustrated by the blue circled region in Fig. 7H, thereby failing to provide accurate target identification. These results underscore the potential impact and effectiveness of our virtual staining technique in reliably localizing and identifying vascular invasion.

## Discussion

Accurate identification of vascular invasion, defined as the presence of tumor cells within blood or lymphatic vessels, is pivotal for determining cancer prognosis and guiding clinical management due to its critical role in facilitating metastatic dissemination. Conventional histopathological assessment typically starts with H&E-stained sections, aimed at concurrently visualizing the entire tissue composition, including tumor cells and vascular structures. However, identifying blood vessels, and specifically, small-caliber vessels, can be confounded by artifacts such as tissue retraction, cracks, or pseudovascular spaces, complicating the reliable distinction between true vascular structures and artifact-induced formations. This leads to a significant number of cases where the vascular lumen cannot be definitely identified in the H&E section. To mitigate these limitations, routine clinical workflows often incorporate additional IHC stains such as ERG to confirm endothelial lining presence and PanCK to validate tumor cell localization within the suspected lumen space, typically performed on serial tissue sections, as illustrated in Fig. 1A. However, this sequential staining process poses challenges, notably the potential loss of critical areas of interest—especially those involving small-caliber vessels—due to tissue dropout or because the examined H&E-stained section represents the final level at which the vessel is still present. Multiplexed staining methods, which enable simultaneous detection of multiple biomarkers, have emerged to directly address multiple antigen localization on the same tissue slide. Nonetheless, none of these advanced methods are incorporated into routine diagnostic workflows as they typically require specialized imaging equipment, complex analytical frameworks, substantial financial resources, and prolonged tissue processing times. Furthermore, multiplexed staining methods are typically performed on sections sequential to the H&E-stained slide. If a vessel is present only on the H&E section and absent at deeper levels, it will not be detected using these methods.

In this study, we introduced a virtual mIHC framework designed to transform label-free AF microscopy images of thyroid tissue sections into BF equivalents of H&E, ERG, and PanCK stains. This framework demonstrated strong concordance with traditional histochemical staining results. Importantly, simultaneous generation of multiple stains (H&E and 2 different IHCs) on a single physical tissue section, as achieved here, is unattainable through current standard histopathological methods, and this capability allows for the definite detection of small-caliber vessels present only on the initial section. Our approach ensures the accurate colocalization of tumor cells and blood vessels within the same physical tissue section, significantly enhancing diagnostic accuracy by eliminating issues such as tissue dropout or the possibility that the examined H&E-stained section represents the last level where the vessel is still visible, thus preserving areas of interest and improving diagnostic efficiency.

Our virtual mIHC method requires only a single tissue section subjected to AF imaging, circumventing the cumbersome and labor-intensive process of handling serial tissue sections and subsequent individual histochemical staining steps. This can streamline clinical workflows and conserve valuable tissue for subsequent advanced molecular analyses. AF microscopy was chosen as the imaging input modality due to its proven effectiveness in multiple virtual staining studies [11,26,27,34,36] and its ease of integration with existing U.S. Food and Drug Administration (FDA)-cleared whole-slide imaging systems [39,40] without additional optical components or significant costs. After a one-time training phase—including data acquisition, preprocessing, and network optimization—our multiplexed virtual staining framework can rapidly generate virtual mIHC images. Specifically, it completes inference in mere seconds for individual fields (e.g., <2 s for a 2,000 × 2,000 pixel field-of-view) and in a few minutes for a whole-slide image when leveraging modest graphics processing units (GPUs).

For the network architecture, we chose a cGAN-based model by design. Recent diffusion [41,42] and visual autoregressive models [43] have achieved exceptional performance in generative settings, where inputs are compromised, e.g., severely undersampled or with much lower resolution, where the network must rely on strong learned priors to extrapolate missing information. In our application, both the AF input and the BF/IHC target images are acquired using diffraction-limited optical systems; therefore, the task is dominated by learning a structurally faithful mapping between 2 well-resolved imaging domains rather than reconstructing information that is, for example, undersampled or compromised. Consistent with this, in our previous work [32] on diffraction-limited, high-resolution virtual staining, we have not observed any systematic advantages of diffusion-based virtual staining architectures over cGANs for reproducing fine histologic details. Considering its speed advantages compared to iterative diffusion models, we consider cGAN-based virtual staining a competitive state-of-the-art method, with demonstrated success in reproducing diagnostically relevant morphology in various types of virtually stained images. Furthermore, models with stronger generative priors, such as diffusion and autoregressive architectures, generally carry an increased risk of hallucinations [44]. In the context of vascular invasion, this could translate into artifactual vascular invasion, such as foci or vascular profiles, or conversely, into subtle loss or smoothing of endothelial linings and intravascular tumor clusters, either of which would materially alter the inferred VI (vascular invasion) status. We therefore adopted a cGAN backbone as a safety-aware architecture that we consider best suited to virtual mIHC in this setting, and that also affords faster single-pass inference, compatible with high-throughput histology workflows.

In this work, we selected thyroid TMAs to demonstrate our multiplexed virtual staining method. The TMAs consist of small cores sampling both neoplastic and non-neoplastic thyroid parenchyma, and many cores contain only limited neoplastic tissue with few or no unequivocal VI foci. Under these conditions, case-level VI status cannot be robustly reconstructed, and the number and spatial extent of VI-positive regions are insufficient to support precise estimates at the patient level. An important next step would be to evaluate our approach in multi-institutional whole-slide cohorts enriched for VI-positive cases, enabling more comprehensive validation of its diagnostic utility and extended characterization of case-level performance and generalizability.

The robustness of our method was validated through a blinded evaluation by 4 board-certified pathologists, confirming that virtually stained ERG and PanCK images accurately highlighted targeted endothelial and epithelial cells, respectively. In the study, we did not focus on H&E image quality comparisons because virtual H&E staining using label-free microscopy (e.g., AF microscopy) and cGANs has been extensively demonstrated to be robust and physiologically equivalent to the histochemical H&E counterparts in previous efforts [11,12,14,15,17,20–22,26,45,46]. Notably, the statistical superiority analysis demonstrated in Figs. 5E and 6E revealed that virtual staining results in general provided superior consistency in staining intensity and specificity. This comparative analysis highlights another significant advantage of our virtual mIHC staining model—its stability and reproducibility—delivering consistently high-quality staining images. Conversely, conventional histochemical staining frequently encounters various technical artifacts, such as uneven staining quality, overstaining, understaining, and pigment deposition, leading to morphological alterations and diagnostic challenges, as illustrated in Fig. 7H. Furthermore, traditional IHC staining sometimes introduces artifacts like nonspecific antibody binding and variability in antibody quality, contributing to inadequate or inconsistent visualization of targeted cells, exemplified by the weaker staining intensity observed in histochemical ERG images (Fig. 5).

Our results, depicted in Fig. 7, further demonstrate that the virtual mIHC approach effectively identifies and localizes vascular invasion. Within the same tissue section, pathologists can initially perform routine cancer assessments using the virtual H&E image to identify suspicious regions. Virtual ERG and PanCK images, at the same nanoscopic grid of the same tissue section, can subsequently highlight endothelial cells around vessels and epithelial cells, respectively, enabling accurate identification of vascular invasion. In contrast, traditional diagnostic workflows risk losing the region of interest due to tissue dropout or the vessel not being present on deeper levels, even after initially pinpointing suspicious areas on H&E-stained sections. Our virtual mIHC staining framework provides a reliable and consistent solution, potentially improving diagnostic accuracy and workflow efficiency.

The integration of a DSM with the virtual staining model ensures internal consistency across the generated staining types, maintaining structural alignment between the virtual mIHC and H&E outputs. Conversely, employing separate neural networks for each virtual stain could introduce divergent artifacts and network-to-network inconsistencies, potentially complicating clinical interpretation. An exciting future research direction could involve automated label-free detection of potential vascular invasion areas, subsequently applying multiplexed microstructured staining, e.g., ERG around blood vessels, PanCK within vessels, and H&E in surrounding regions. Such multiplexed microstructured staining could further assist pathologists by accelerating diagnostic workflows.

An analysis of incorrect predictions revealed 2 primary patterns of failure. First, suboptimal input quality—particularly defocused AF image acquisition—led to the attenuation of fine structural contrast, resulting in reduced fidelity of the corresponding virtual stains, as shown in Fig. S2A. Because the model relies on high-frequency morphological cues to infer structural boundaries and stain localization, loss of focus directly propagates to weaker predictions. This limitation reflects image acquisition-driven variability rather than algorithmic instability and can be mitigated through the enforcement of autofocus, quality-control filtering, and the exclusion of poorly focused image regions during training and testing. Second, as illustrated in Fig. S2B, false-positive PanCK signals arose from the rare presence of high endothelial venules (HEVs), whose plump, cuboidal endothelial nuclei closely mimic epithelial cell morphology. This biological confounder represents a localized class imbalance rather than a systemic failure of stain differentiation and is consistent with known diagnostic challenges even under conventional IHC evaluation. Importantly, these errors were restricted to isolated foci and did not affect broader diagnostic regions. Future enhancements, such as selectively incorporating HEV-rich regions into training, applying class-weighted optimization, or hard-example mining, are expected to reduce the number of false positive regions. Together, these observations clarify the underlying causes of observed failure modes and highlight pathways toward improving robustness.

While this work demonstrates strong concordance and diagnostic interpretability of virtual multiplex staining, our current validation was performed primarily on thyroid TMAs sourced from a single site and acquired using a uniform AF imaging setup. This focused effort enabled rigorous control of staining quality, sectioning consistency, and ground-truth evaluation, allowing for a clear demonstration of the feasibility and diagnostic value of simultaneously generating virtual H&E, ERG, and PanCK images on the same physical section. Nonetheless, acquiring broader evidence across additional tissue types, multi-site cohorts, and routine surgical resections remains an important next step to characterize generalizability fully. Additionally, although AF imaging served effectively as the input modality in this study, AF is not currently an FDA-cleared pathology image acquisition method; therefore, the present results should be interpreted as establishing the technical capability rather than implying regulatory status. Future work will focus on generating larger and more diverse datasets and evaluating cross-site performance, which would further help characterize generalizability and strengthen the translational potential of the proposed framework.

In conclusion, our virtual mIHC method effectively transforms label-free thyroid tissue sections into BF microscopy images equivalent to histochemical H&E, ERG, and PanCK stains. This method facilitates rapid and accurate localization and identification of vascular invasion, eliminating the need for IHC staining procedures on serial tissue sections and reducing the risk of losing areas of interest due to tissue dropout or heterogeneity. Our approach holds promise for significantly transforming conventional diagnostic workflows used for vascular invasion, paving the way for extensive, multi-center clinical validation studies to further advance the clinical translation and adoption of the presented technology.

## Methods

### Sample preparation, image acquisition, and histochemical staining

Ten unlabeled, anonymized thyroid TMA slides, each containing ~80 cores, were acquired from TissueArray [47]. Following AF imaging, 2 slides were sent for standard H&E staining, 3 for IHC PanCK staining, and 4 for ERG staining. All histochemical staining procedures were performed at the University of Southern California (USC) Pathology Lab. The AF images of label-free thyroid core biopsies were captured using a Leica DMI8 microscope with a 40×/0.95 numerical aperture (NA) objective lens (Leica HC PL APO 40×/0.95 DRY), controlled using Leica LAS X microscopy automation software. Four fluorescence filter cubes, including 4′,6-diamidino-2-phenylindole (DAPI) (Semrock OSFI3-DAPI5060C, excitation 377/50 nm, emission 447/60 nm), TxRed (Semrock OSFI3-TXRED-4040C, excitation 562/40 nm, emission 624/40 nm), fluorescein isothiocyanate (FITC) (Semrock FITC-2024B-OFX, excitation 485/20 nm, emission 522/24 nm), and Cy5 (Semrock CY5-4040C-OFX, excitation 628/40 nm, emission 692/40 nm), were used to acquire the 4 AF image channels. This image acquisition was completed using a scientific complementary metal-oxide semiconductor (sCMOS) image sensor (Leica DFC 9000 GTC) with exposure times of 150, 300, 300, and 500 ms for the DAPI, FITC, TxRed, and Cy5 filters, respectively. After the histochemical staining process, the tissue samples were imaged using a pathology scanner (Aperio AT2, Leica Biosystems, 20×/0.75 NA objective with a 2× adapter) for capturing the ground-truth BF images.

### Image preprocessing and pretraining registration

To enable supervised learning for virtual staining, precise alignment between the AF images and their corresponding BF ground-truth images was required. As summarized in Fig. S3, our framework incorporates 2 complementary alignment stages: (a) pretraining registration for generating well-aligned AF–BF data pairs and (b) registration refinement applied during the model training. Figure S3A illustrates the pretraining registration workflow. First, we stitched all the scanned images into the whole-slide images, including TMA core biopsies for AF and BF imaging modalities. Subsequently, image pairs of each core were globally registered using a multi-modal registration algorithm [48,49]. Second, these roughly aligned AF and BF images for core biopsies were divided into small image tiles with 2,000 × 2,000 pixels. Finally, our data processing includes a correlation-based elastic registration step, which is critical to further match the image pairs with the local and global corrections and address optical aberrations of different imaging modalities and morphological distortions resulting from histochemical staining procedures. During this step, a vanilla virtual staining network was first trained to translate the AF images into the BF-stained versions (PanCK, ERG, and H&E) as well as elastically align BF images to the AF images during the training process [26]. This vanilla virtual staining network module shared the same architecture and settings as our virtual staining network (see the “Neural network architecture” section). We further aligned the BF images into the style-transferred images (obtained using AF images) by using the pyramid elastic registration algorithm [11,50]. This algorithm includes pyramidal matching of local features across multi-resolution sub-image blocks to calculate transformation maps and then correction of the local distortions related to aberrations and staining procedures. This elastic registration step was repeated until very well-registered image pairs were obtained to enable accurate training of the virtual staining model. These registration algorithms were implemented using MATLAB (MathWorks) and Python.

To account for residual spatial deviations that may persist even after preprocessing, model training incorporated a registration module to ensure that pixel-wise supervision is applied to spatially aligned image pairs, as illustrated in Fig. S3B. This mechanism enables consistent spatial correspondence during loss computation, thereby improving training robustness. The detailed implementation of this registration module and its role in optimization are described in the “Neural network architecture” section.

### Dataset division and preparation

After image processing and registration, we obtained 3 datasets consisting of paired AF images and their corresponding histochemically stained counterparts (PanCK, ERG, and H&E). For the H&E dataset, we used 4,416 image pairs (2,000 × 2,000 pixels for each tile) from 140 TMA cores for training and reserved 12 whole TMA cores (both AF and H&E-stained) for blind testing. For the PanCK dataset, we used 3,598 image pairs from 146 TMA cores for training and reserved 12 whole TMA cores for testing. For the ERG dataset, we used 1,216 image pairs from 70 TMA cores for training and reserved 6 whole TMA cores for testing. Due to technical limitations inherent to histochemical ERG staining—such as suboptimal antibody performance leading to poor visualization of endothelial cells—many ERG-stained cores with insufficient endothelial cell visibility were excluded from our ground-truth images based on the evaluation of a board-certified pathologist (N.P.). As a result, the ERG staining dataset was approximately half the size of the H&E and PanCK datasets due to the relatively poor repeatability of the ERG histochemical staining. To address this class imbalance during training, we designed the data pipeline to ensure equal representation: In each batch, image pairs were randomly sampled from the 3 staining datasets with equal probability.

### Neural network architecture

**Fig. 8.**
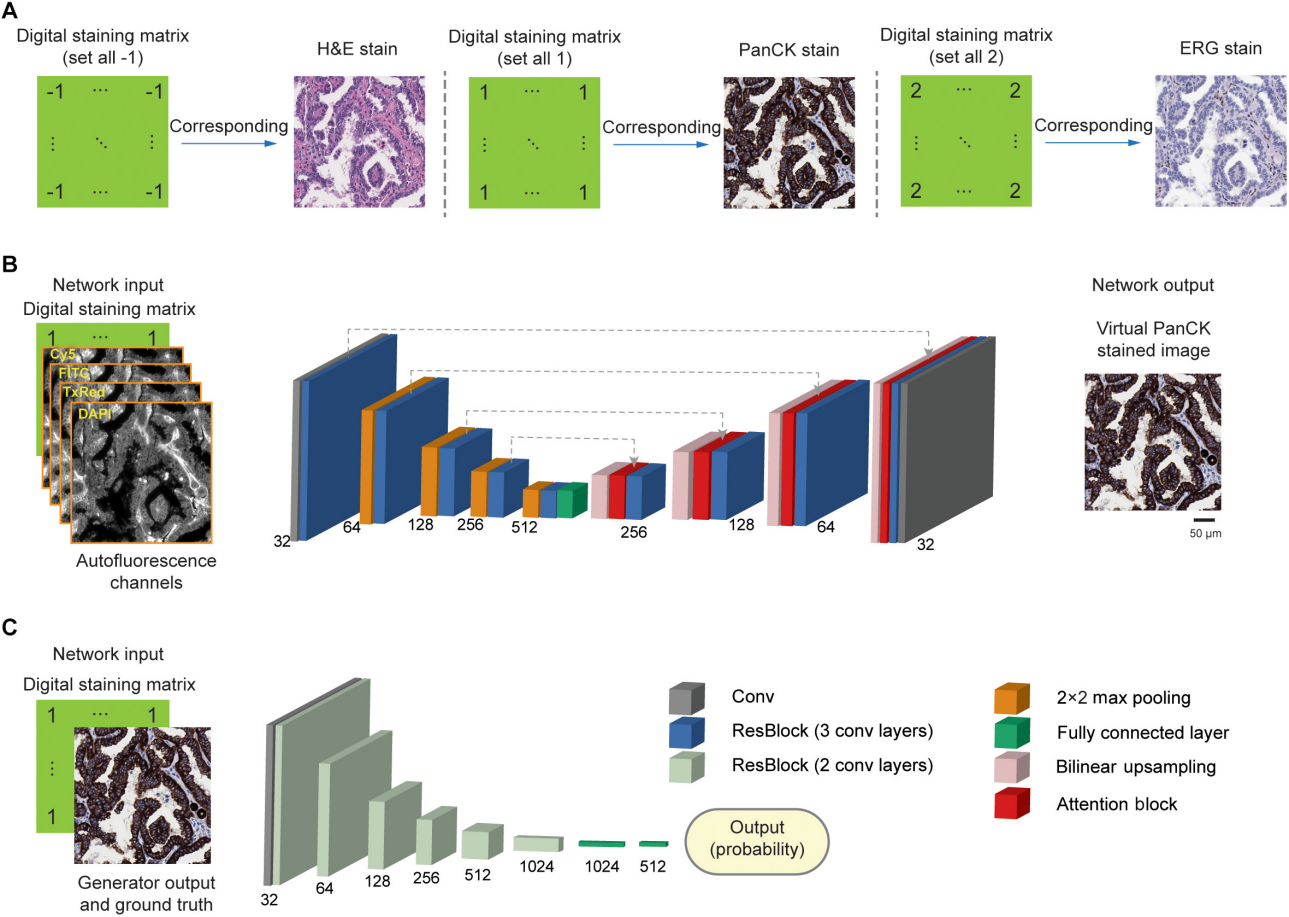
Network architecture of the virtual mIHC staining model. (A) DSM configurations used during both the training and testing phases to generate multiplexed virtual stains: all elements set to −1 for H&E, 1 for PanCK, and 2 for ERG. (B) Detailed architecture and building blocks of the generator. (C) Detailed architecture and building blocks of the discriminator.

We used a cGAN architecture [51,52] to design a virtual mIHC staining model that rapidly transforms label-free AF images (DAPI, FITC, TxRed, and Cy5) of tissue samples into their corresponding BF images for ERG, PanCK, and H&E virtual staining. The cGAN architecture includes 2 subnetworks, namely, a generator (*G*) and a discriminator (*D*). To enable the multiplexing feature for this virtual staining model, a DSM is concatenated with the label-free input images. As shown in Fig. 8A, the DSM $\tilde{c}\;$is generated using 1 of 3 labels $\tilde{c}=\left[-1\right]\;\text{or}\;\left[1\right]\;\text{or}\;\left[2\right]$, which correspond to different virtual staining tasks, i.e., virtual ERG, PanCK, and H&E staining, respectively. For example, $\tilde{c}=\left[-1\right]$ denotes a DSM for ERG with all elements equal to −1. During the training phase, the generator was optimized to learn the virtual multiplexed staining process. In the meantime, the discriminator was trained to separate the generator outputs from ground-truth stained images. The generator and the discriminator networks were trained using the following loss functions:

lgenerator=αLφItargetGIinputc~∘RGIinputc~Itarget+βBCEDGIinputc~c~1+γTVGIinputc~ (1)

$$l_{\text{discriminator}}=\mathrm{BCE}\left\{D\left(G\left(I_{\text{input}},\;\tilde{c}\right),\;\tilde{c}\right),\;0\right\}+\mathrm{BCE}\left\{D\left(I_{\text{target}},\;\tilde{c}\right),\;1\right\}$$ (2)

where $G\left(\cdot \right)$ and $D\left(\cdot \right)$ refer to the outputs of the generator and discriminator networks, respectively. $I_{\text{target}}$ denotes the ground-truth images, and $I_{\text{input}}$ denotes the input label-free AF images. The binary cross-entropy loss (BCE) is defined as follows:

$$\text{BCE}\left(p\left(a\right),\;p\left(b\right)\right)=-\left[p\left(b\right)\ln\left(p\left(a\right)\right)+\left(1-p\left(b\right)\right)ln\left(1-p\left(a\right)\right)\right]$$ (3)

where $p\left(a\right)$ represents the discriminator prediction after a sigmoid operation and $p\left(b\right)$ represents the actual label (0 or 1). The total variation (TV) loss acted as a regularizer term and was defined as follows:

$$\begin{array}{c} \text{TV}\left\{I\right\}=\sum_{n}\sum_{m}\left|I_{n+1,m}-I_{n,m}\right|+\left|I_{n,m+1}-I_{n,m}\right| \end{array}$$ (4)

where n and m are the pixel indices of the image I. The coefficients $\left(\alpha ,\;\beta ,\;\gamma \right)$ in Eq. (1) were empirically set to $\left(10,1,0.0001\right)$. $R\left(\cdot \right)$ in Eq. (1) refers to an image registration network that was only used during the training phase. As shown in Fig. S3B, it learns to remove alignment errors between virtual staining image outputs and the target (ground truth) images and helps to optimize more accurate virtual staining models. $R\left(\cdot \right)$ takes generator image outputs and the corresponding target images and provides a displacement matrix, aligning virtually stained fields of view with the ground truth. This operation is denoted using “∘”. The loss function of this registration module was defined as follows:

$$\begin{array}{c} \begin{array}{l} l_{\text{registration}}=\lambda \times L_{\varphi }\left\{I_{\text{target}},\;G\left(I_{\text{input}},\;\tilde{c}\right)\circ R\left(G\left(I_{\text{input}},\;\tilde{c}\right),\;I_{\text{target}}\right)\right\}\\ \;+\mu \times \text{SMTH}\left\{R\left(G\left(I_{\text{input}},\;\tilde{c}\right),\;I_{\text{target}}\right)\right\} \end{array} \end{array}$$ (5)

where the smooth loss (SMTH) [53] is defined as:

$$\text{SMTH}\left\{T\right\}=\frac{1}{\mathrm{XY}}\sum_{x}\sum_{y}{\left|T_{x+1,y}-T_{x,y}\right|}^{2}+{\left|T_{x,y+1}-T_{x,y}\right|}^{2}$$ (6)

where T is the displacement matrix. XY denotes the total number of elements in the matrix; x and y are the element indices. The coefficients $\left(\lambda ,\mu \right)$ were empirically set as $\left(20,10\right)$. $L_{\varphi }$ loss, also known as the Huber loss [54], is defined as follows:

LφAB=1MNΣAmn−Bmn<φAmn−Bmn22φ+ΣAmn−Bmn≥φAmn−Bmn−0.5φ     (7)

where m and n denote the pixel indices of images *A* and *B*, and MN denotes the total number of pixels in each image. φ was set to 1.

It is important to note that the registration module R is different from the vanilla virtual staining network used in the pretraining registration steps. Here, the registration module R was used to correct global and local misalignments, which are due to optical aberrations and the chemical staining process, during the training stage to learn more accurate virtual staining models. As described in Eq. (5) and illustrated in Fig. S3B, the registration module warped the network’s in-training output to match the target image, ensuring that the pixel-wise loss was computed correctly, effectively regularizing the training. However, during the data preprocessing steps, the pretraining registration operated differently: It distorted the ground-truth images to generate well-aligned AF–BF image pairs for the subsequent training process (as shown in Fig. S3A).

The generator network (see Fig. 8B), based on an Attention U-Net architecture [55], features a symmetrical encoder–decoder structure. The encoder path comprises 4 downsampling blocks, each containing a 3-convolutional-layer residual block [56] (formed by 3 consecutive convolutional layers and a convolutional residual path), followed by a Leaky ReLU [57] activation (slope 0.1) and a 2 × 2 max pooling layer (stride 2) that halves spatial dimensions and doubles channel depth. Conversely, the decoder path consists of 4 upsampling blocks. Input to each upsampling block is a concatenation of the previous upsampled output and corresponding feature maps from the encoder, which are first processed by an attention gate (3 convolutional layers and a sigmoid operation) to highlight salient features. These upsampling blocks then perform 2× bilinear resizing and utilize a 3-convolutional-layer residual block to reduce channel count by a factor of 4. The network concludes with the final upsampling block, followed by another 3-convolutional-layer residual block and a single convolutional layer, ultimately reducing the channels to 3 to match the ground-truth image format.

The discriminator network, as shown in Fig. 8C, is designed to distinguish between the virtually stained images and the actual ground-truth images. It begins by processing an input image through a single convolutional layer to create a 64-channel tensor, immediately followed by a Leaky ReLU activation function. This tensor then passes through 5 successive residual blocks, each composed of 2 convolutional layers. Within each block, the second convolutional layer uses a stride of 2, which achieves 2× downsampling of the spatial dimensions while simultaneously doubling the number of feature channels. After these feature extraction blocks, a global pooling layer aggregates the learned features, which are then fed into 2 dense (fully connected) layers to ultimately produce a probability score indicating whether the input image is likely a real ground-truth image or not.

The registration module [58] is built upon a U-Net architecture, similar to the generator but with key differences. It features 7 pairs of downsampling and corresponding upsampling blocks, with each block in both paths being integrated with a residual block to facilitate feature propagation. At the deepest point of the network, following the final downsampling stage, a convolutional layer doubles the feature channels. This is succeeded by a series of 3 consecutive residual blocks for further feature refinement. Subsequently, another convolutional layer halves the channel count, preparing the features for the upsampling sequence. The network culminates in an output layer that utilizes a single convolutional layer to condense the feature channels to 2, directly corresponding to the 2 normal components of the displacement matrix it aims to predict. This registration module, together with the discriminator, was only used during the training phase to learn more accurate virtual staining models.

Image patches with 512 × 512 pixels were used during the training of the models, which were obtained by randomly cropping the training images. Also, several data augmentation methods, including random image rotations (0°, 90°, 180°, and 270°) and random flipping, were applied to these image patches. Adam optimizer [59] was used to optimize the generator, discriminator, and registration modules (with the learning rates of $2\times 10^{-5}$, $2\times 10^{-6}$, and $2\times 10^{-6}$, respectively). The batch size was set to 4. The generator parameters were updated 4 times for each update of the discriminator parameters. The training duration of the virtual staining models was approximately 72 h. A computer with GeForce RTX 3090 Ti GPUs, 256 GB of random-access memory (RAM), and an Intel Core i9 central processing unit (CPU) was used during training and blind evaluation of the models. These deep neural network models were implemented using Python version 3.12.0 and PyTorch [60] version 1.9.0 with CUDA toolkit version 11.8.

### Quantitative quality evaluation of virtual H&E, ERG, and PanCK staining

To quantitatively assess the fidelity of the virtually stained H&E, ERG, and PanCK images generated by our neural network model against their histochemically stained ground truths, we first employed 3 standard metrics: PSNR, SSIM [37], and LPIPS [38]. The evaluation dataset comprised 390 virtual-histochemical H&E image pairs from 12 TMA cores, 197 ERG pairs from 6 TMA cores, and 149 PanCK pairs from another 6 TMA cores.

The PSNR is defined as follows:

$$\text{PSNR}\left(I_{\mathrm{VS}},\;I_{\mathrm{HS}}\right)=10\;\text{lg}\left(\frac{\text{max}{\left(I_{\mathrm{HS}}\right)}^{2}}{\mathrm{MSE}\left(I_{\mathrm{VS}},\;I_{\mathrm{HS}}\right)}\right)$$ (8)

where $I_{\mathrm{VS}}$ presents the virtually stained image, $I_{\mathrm{HS}}$ stands for its histochemically stained counterpart, and MSE is the mean squared error defined as follows:

$$\mathrm{MSE}\left(I_{\mathrm{VS}},\;I_{\mathrm{HS}}\right)=\frac{1}{W\times H}\sum_{i}\sum_{j}{\left[I_{\mathrm{VS}}\left(i,\;j\right)-I_{\mathrm{HS}}\left(i,\;j\right)\right]}^{2}$$ (9)

where *i* and *j* represent the pixel indices, and *W* and *H* refer to the image width and height, respectively.

While PSNR and SSIM measure low-level signal fidelity, LPIPS evaluates perceptual similarity by measuring the distance between image patches in deep feature space, a method shown to align closely with human visual judgment. We utilized the standard LPIPS implementation with an AlexNet backbone pretrained on ImageNet, calculating the weighted Euclidean distance between feature maps of the generated virtually stained output and its corresponding ground truth across multiple network layers.

In addition to these standard metrics, which are widely used in the computer vision area, we also analyzed diagnostic features specific to ERG and PanCK stains. We first applied a color deconvolution method [61] to isolate the DAB channel (shown as the brown chromogen) from the hematoxylin background. For ERG images, Otsu’s thresholding [62] followed by morphological operations (dilation and erosion) was applied to the DAB channel to generate binary masks of ERG-positive nuclei, as demonstrated in Fig. S4A. We quantified the nuclei count and the average nuclear area (measured in pixels^2^) for each field-of-view, defined as the count and average area of connected components in the ERG-positive binary mask. To determine statistical significance, paired 2-tailed *t* tests were performed on the nuclei count and average area metrics comparing the virtually and histochemically stained images.

For PanCK images, Otsu’s thresholding was similarly applied to the DAB channel to extract masks of the highlighted epithelial cells, as shown in Fig. S4B. To evaluate the spatial overlap between the virtually stained and histochemically stained DAB masks, D-IoU [63] was calculated as follows:

$$D-\mathrm{IoU}=\frac{\sum_{i,j}\left(M_{\mathrm{VS}}^{↓}*M_{\mathrm{HS}}^{↓}\right)}{\sum_{i,j}\min\left(\left(M_{\mathrm{VS}}^{↓}+M_{\mathrm{HS}}^{↓}\right),1\right)}$$ (10)

where $M_{\mathrm{VS}}^{↓}$ and $M_{\mathrm{HS}}^{↓}$ represent the 8× down-sampled binary masks of the PanCK-positive epithelial cell regions for the virtual and histochemical images, respectively. D-IoU was employed here, instead of traditional per-pixel IoU, to ensure robustness against minor pixel-level misalignments and to assess similarity from a macroscopic structural perspective. ∗ and + denote pixel-wise multiplication and addition, respectively, and $\left(i,j\right)$ denotes the pixel indices. Prior to calculating D-IoU, the down-sampled masks were binarized using a threshold of 0.01.

## Ethical Approval

We acquired deidentified, existing specimens from TissueArray [47] collected before and independent of this work, with no patient information that can be linked through the image. These deidentified images/samples are not used for clinical diagnosis or as a replacement for the regular clinical workflow.

## Data Availability

Deep learning models reported in this work used standard libraries and scripts that are publicly available in PyTorch. The authors declare that all data supporting the results of this study are available within the main text. Anonymized TMA slides (from existing specimens) were acquired from TissueArray [47]. The testing data are archived in Zenodo and are accessible via https://doi.org/10.5281/zenodo.17924856 [64].
