# Supplementary material for: Deep Learning-Enabled Virtual Multiplexed Immunostaining of Label-Free Tissue for Vascular Invasion Assessment
Source: BME Front. 2026 Feb 10;7:0226. doi: 10.34133/bmef.0226 (PMC12886716; doi:10.34133/bmef.0226)
Supplement: Supplementary 1 — Figs. S1 to S4 [file bmef.0226.f1.pdf]

Supplementary Information for

Deep learning-enabled virtual multiplexed immunostaining of label-free tissue for vascular invasion assessment

Yijie Zhang[†,1,2,3], Çağatay Işıl[†,1,2,3], Xilin Yang[1,2,3], Yuzhu Li[1,2,3], Anna Elia[4], Karin Atlan[4], William Dean Wallace[5], Nir Pillar[*,1,2,3,4], and Aydogan Ozcan[*,1,2,3,6]

[1]Electrical and Computer Engineering Department, University of California, Los Angeles, CA, 90095, USA.

[2]Bioengineering Department, University of California, Los Angeles, CA, 90095, USA.

[3]California NanoSystems Institute (CNSI), University of California, Los Angeles, CA, 90095, USA.

[4]Department of Pathology, Hadassah Hebrew University Medical Center, Jerusalem, 91120, Israel

[5]Department of Pathology, Keck School of Medicine, University of Southern California, Los Angeles, CA, 90033, USA.

[6]Department of Surgery, University of California, Los Angeles, CA, 90095, USA.

*Correspondence: Nir Pillar nir.pillar@mail.huji.ac.il, Aydogan Ozcan, ozcan@ucla.edu

[†] Equal contributing authors

Supplementary Figures:

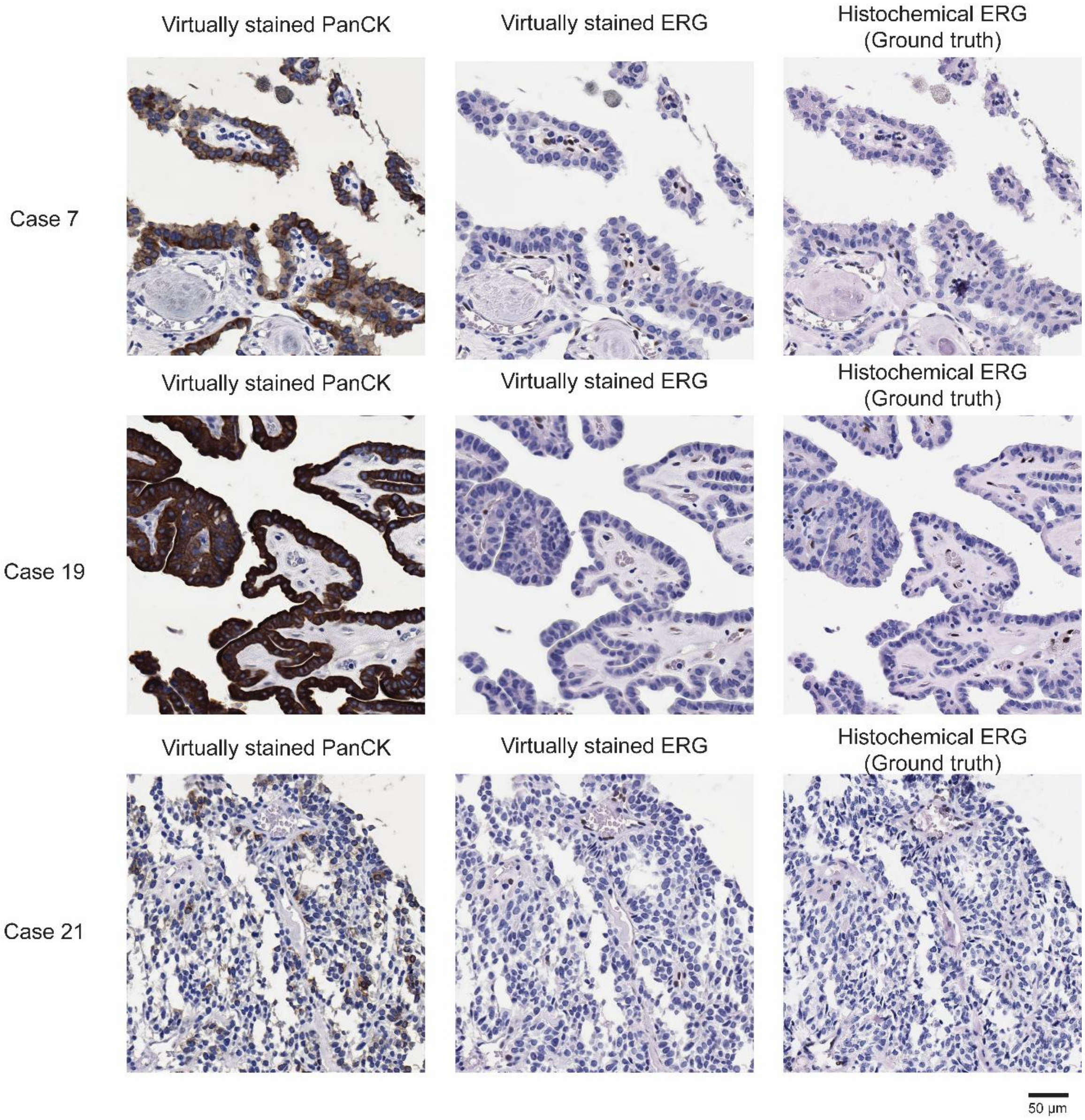

Supplementary Figure 1. Representative case study tiles for pathological evaluation. Virtual PanCK and ERG images alongside the corresponding histochemical ground truth for cases #7, #19, and #21 referenced in Figure 4 of the main text.

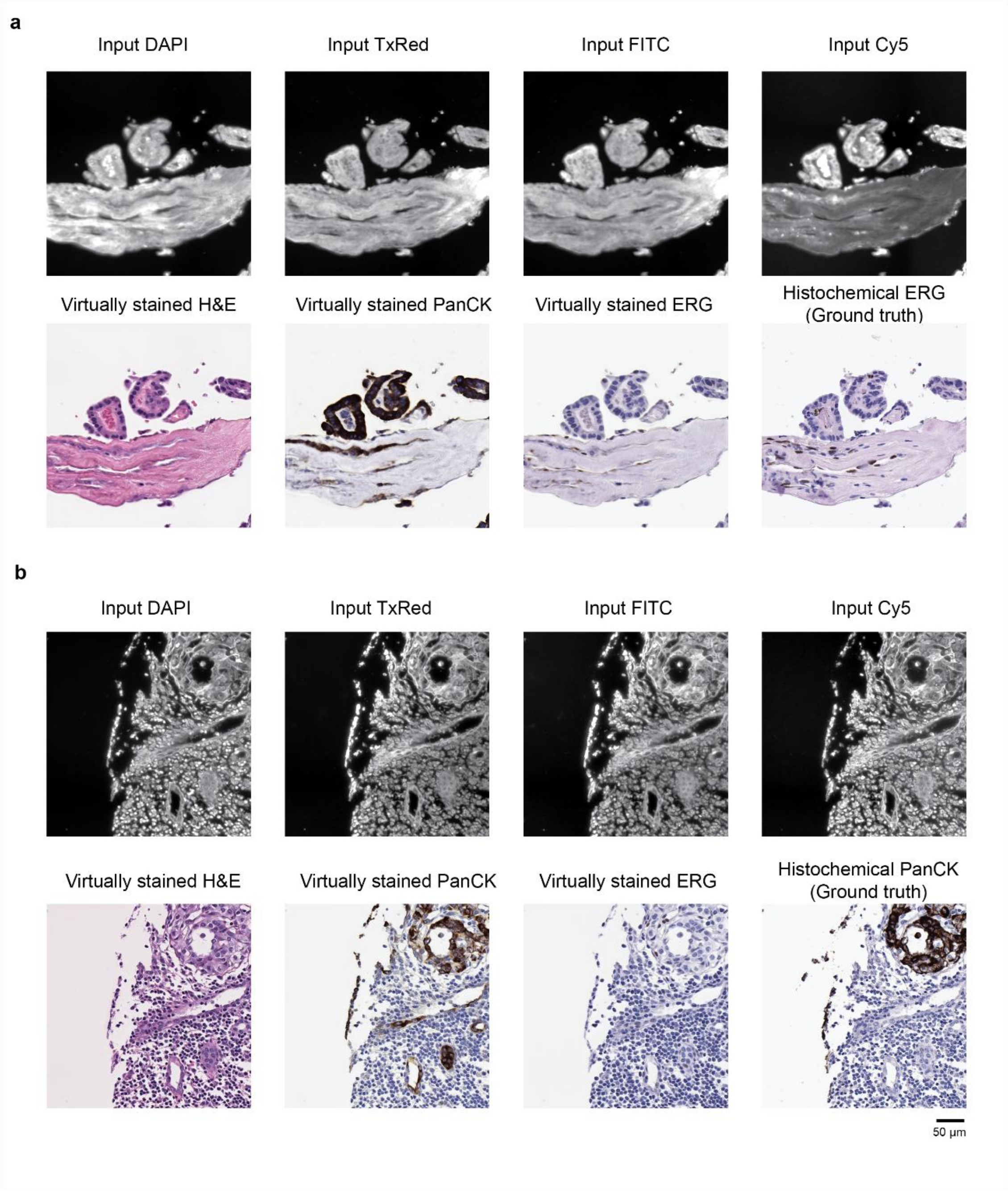

Supplementary Figure 2. Examples of two failure cases. The input (four) autofluorescence channels and the three virtual stains, and the corresponding ground truth images are presented. (a) A failure case caused by the input autofluorescence image being out of focus. (b) A failure case of rare presence of high endothelial venules (HEVs), representing a challenging negative due to morphological mimicry.

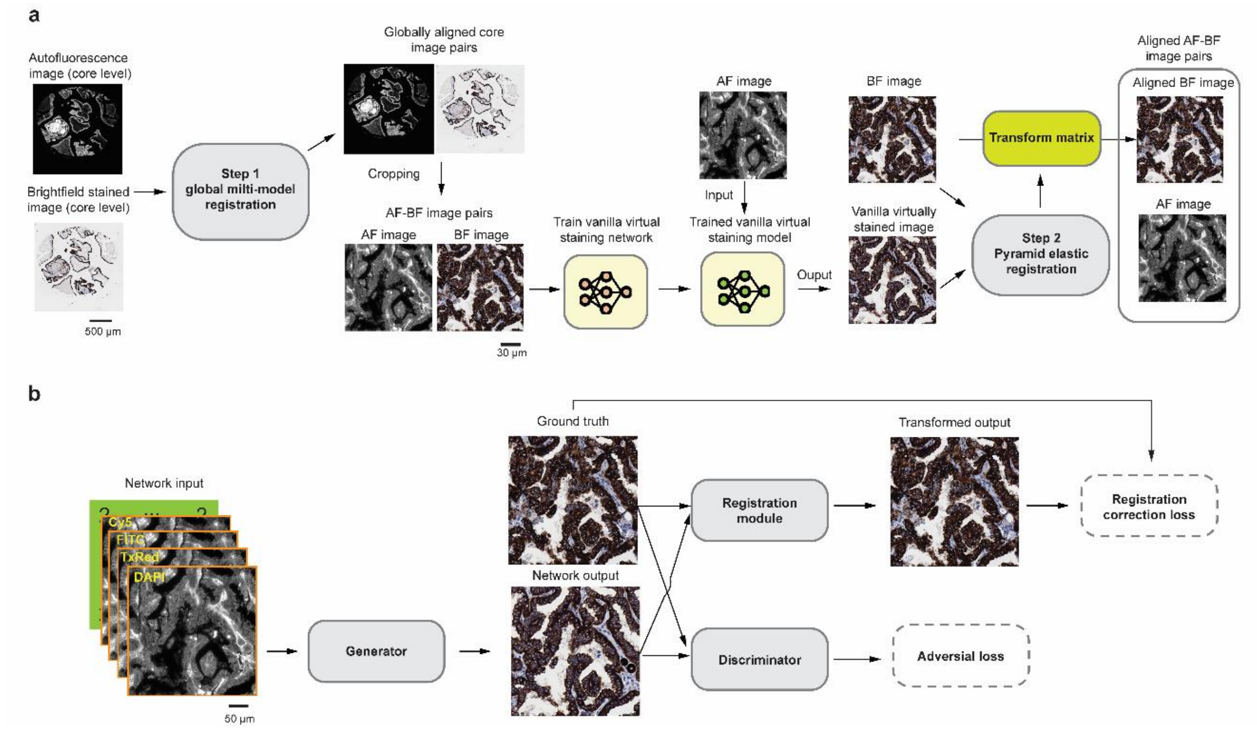

Supplementary Figure 3. Registration pipeline. (a) Pre-training registration workflow. The procedure begins with global multi-modal registration between autofluorescence (AF) and brightfield (BF) core-level images. The globally aligned AF-BF cores were cropped into fixed-size image pairs (2000 × 2000 pixels), which were then used to train a vanilla virtual-staining network to generate preliminary virtually stained images from AF inputs. A pyramid elastic registration algorithm was subsequently applied to register the BF image to the vanilla virtually stained output, thereby indirectly aligning the BF image with the AF input and producing the final aligned AF-BF training pairs. (b) Registration during the model training. During each training iteration, the network generator output was spatially transformed using the transformation predicted by the registration module. The transformed output was then compared with the ground truth image to compute a registration-correction loss, which jointly optimizes the registration module along with the generator network.

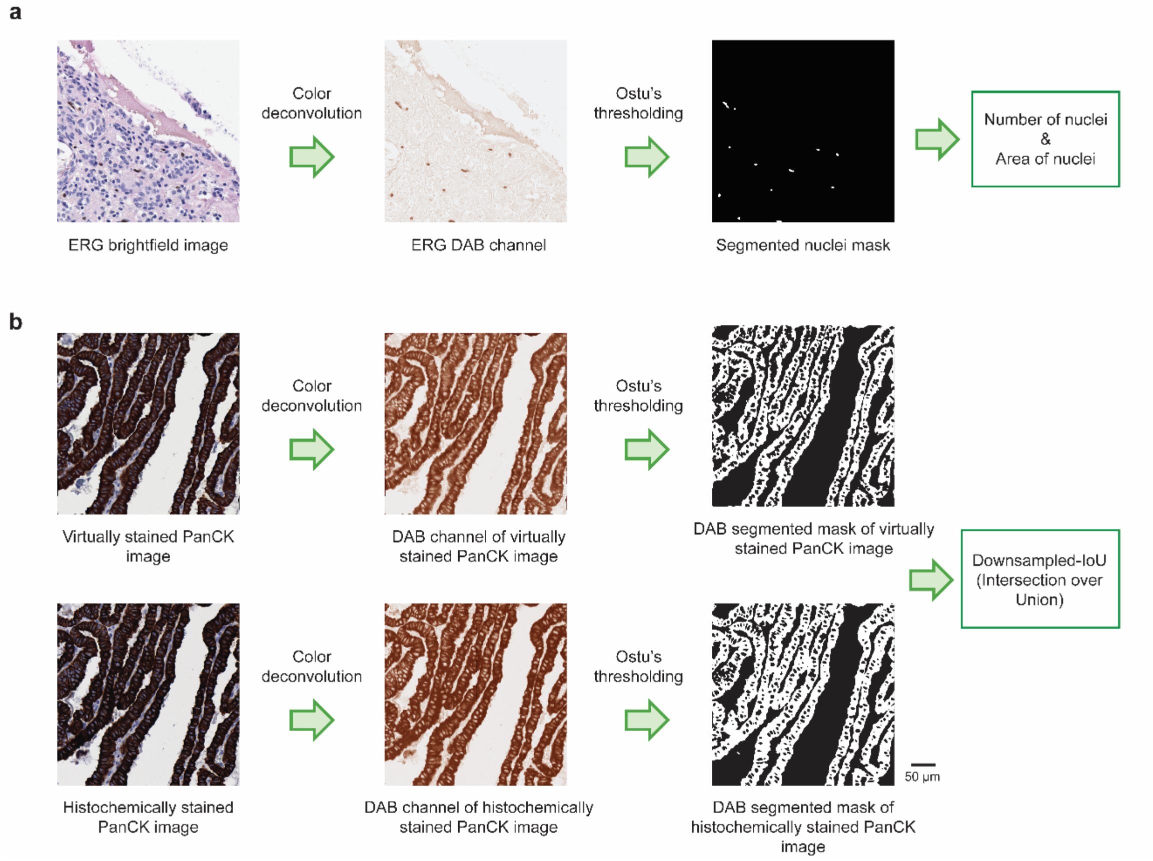

Supplementary Figure 4. Workflow for ERG and PanCK task-specific quantitative evaluation. (a) ERG nuclear localization analysis. Starting from the histochemically stained ERG brightfield image, the DAB channel (brown chromogen) was isolated using color deconvolution. Otsu's thresholding was then applied to the DAB channel to obtain a binary mask of ERG-positive nuclei. The connected components in this mask were used to compute the nuclei count and average nuclear area for each field of view. (b) PanCK epithelial region analysis. For both virtually stained and histochemically stained PanCK images, color deconvolution was performed to extract the DAB channel, followed by Otsu's thresholding to obtain cytoplasmic region masks. These binary masks were compared using the Downsampled Intersection-over-Union (D-IoU) metric, calculated from 8× downsampled masks to provide robustness against minor pixel-level misalignments.
